# Determinants of postnatal care utilization in sub-Saharan Africa: a meta and multilevel analysis of data from 36 sub-Saharan countries

**DOI:** 10.1186/s13052-020-00944-y

**Published:** 2020-11-27

**Authors:** Zemenu Tadesse Tessema, Lake Yazachew, Getayeneh Antehunegn Tesema, Achamyeleh Birhanu Teshale

**Affiliations:** 1 Department of Epidemiology and Biostatistics, Institute of Public Health, College of Medicine and Health Sciences, University of Gondar, Gondar, Ethiopia; 2 Department of Health Systems and Policy, Institute of Public Health, College of Medicine and Health Sciences, University of Gondar, Gondar, Ethiopia

**Keywords:** Postnatal care, Sub-Saharan Africa, Multilevel, Determinants

## Abstract

**Introduction:**

Globally, over 65% of maternal deaths occur during the first 42 days of postpartum while the same proportion of neonatal deaths occur during the first 7 days of life. In sab- Saharan Africa, 4.7 million mothers, newborns, and children die on annual basis. As to our knowledge, there is no study on postnatal care utilization that incorporates all sub-Saharan Africa countries that had DHS data. Therefore, this study aimed at identifying pooled magnitude and determinants of postnatal care utilization in sub-Saharan Africa.

**Method:**

A population-based cross-sectional study from the most recent Demographic and Health Surveys data from the period of 2006 to 2018 of 36 SSA countries were used. A total weighted sample of 286,255 reproductive-age women who gave birth 5 years preceding the survey were included in the study. A meta-analysis of DHS data of each Sub-Saharan countries was conducted to generate pooled magnitude and a forest plot was used to present it. A multilevel logistic regression model was fitted to identify determinants of postnatal care utilization. The AOR (Adjusted Odds Ratio) with their 95% CI and *p*-value ≤0.05 was used to declare that determinates associated with postnatal care utilization.

**Result:**

The pooled magnitude of postnatal care utilization in sub-Saharan Africa countries was 52.48% [95% CI: 52.33, 52.63], with the highest postnatal care utilization in the Central Region of Africa (73.51%) and the low postnatal care utilization in Eastern Regions of Africa (31.71%). In the multilevel logistic regression model region, residence, age group, maternal education, maternal occupation, media exposure, ANC visit, place of delivery, and accessing health care were determinants of postnatal care utilization in Sub-Saharan Africa.

**Conclusion:**

The coverage of postnatal care service utilization was low with high disparities among the region. Being in rural residence, young age group, low education level, had no occupation, not exposed to media, a big problem to access health care, not had ANC visit, and home delivery was associated with low postnatal care service utilization. This study evidenced that there is a wide gap in postnatal care utilization between SSA countries. Special attention is required to improve health accessibility, utilization, and quality of maternal health services to increase postnatal care service utilization in the region.

## Introduction

Our world health systems are challenged with sustained and major maternal mortality. According to the World Health Organization (WHO), 289,000 women die from complications related to pregnancy, labor and delivery, and the postpartum period every year globally [1, 2]. On the other hand, over 1 million neonates die soon after birth every year worldwide, making the day of birth the most critical day for neonates all over the world [3, 4].

Over 65% of maternal deaths occur during the first 42 days of postpartum while the same proportion of neonatal deaths occur during the first 7 days of life. However, almost all (99%) of these maternal and neonatal deaths occur in developing countries with the highest deaths in south Asia and sub-Saharan Africa. In developing countries, maternal death was 15 times higher than in developed countries. The highest maternal and child deaths take place in sub-Saharan Africa, 500 maternal deaths per 100,000 live births every year [1, 2, 5].

In sab Saharan Africa, mothers are more likely to die due to pregnancy, delivery, and post-delivery related conditions. Moreover, 4.7 million mothers, newborns, and children die on annual basis in sub-Saharan Africa: pregnancy and delivery-initiated complications account for 265,000 maternal deaths and 3,192,000 child deaths who are between the age of 1 month and 5 years [3, 4, 6, 7].

Maternal health service utilization is an effective intervention to overcome maternal and child health-related complications. Likewise, postnatal care service is a basic component of the continuum of maternal health services. According to WHO a postnatal period is defined as the time from an hour after the delivery of the placenta through 6 weeks after the delivery of a child. Postnatal care (PNC) comprises of services given to mothers and neonates right after delivery and up to 42 days of postpartum to ensure optimum health for the mother and her infant [5, 8].

Worldwide, PNC has been declared to be a vital maternal health service to keep and promote the health and long life of a mother and newborn. Furthermore, health experts have a chance to detect, follow, and handle the health conditions of both the mother and newborn during postnatal service [9]. Also, post-natal services are the key strategies to prevent the emerging of physical and mental deterioration among post-natal mothers [6].

Following tremendous efforts, antenatal care (ANC) has shown an advanced change across years, while PNC still leftovers below the tolerable speed of improvement in many developing countries. Understanding the role of PNC in the continuum of maternal care during the post-natal period made implementation of policies that intended to improve maternal and newborn health [8].

The postpartum period is a life-threatening time for both mothers and newborns. It is also a time to occur most clinically important changes in the mother’s and newborns’ bodies. Especially, in developing countries [4], PNC is a key maternal health service in reducing maternal morbidity and mortality including sab Saharan Africa. Fail to use this service may bring avoidable deaths and sequelae as well as missed opportunities to promote maternal and child health [10].

Hence, WHO recommends that, when labour is attended in a health facility, for a simple vaginal delivery, ordinary newborns must be given a minimum of 24 h care. Whereas, if the birth happens out of health facility, the first postnatal visit must be within the first 24 h of delivery. Irrespective of the place of delivery, at least three other postnatal visits are arranged for all mothers and newborns, on day 3 (48–72 h), between the 1st and 2nd weeks, and 6 weeks after delivery. Nevertheless, less than a quarter of newborns in less developed countries receive PNC within 48 h of delivery [4].

According to UNICEF’s brief report of 2019, 63% of mothers and just 48% of newborns worldwide received a post-natal health check within the prescribed timeframe [11]. However, in Africa, health institutions are not visited by most women and newborns after birth. This implies that post-natal services are among neglected agendas than all other reproductive and child health programs. The magnitude of postnatal care service utilization is very low, for instance, 47% in Kenya, 41.2% in Nigeria, 43.53% in Tanzania, 43, 55% in Zambia, 57.5% in Ethiopia, and it is highly variable across sab Saharan countries and unconvincing for interventions [4, 10, 12].

Evidence showed that PNC services utilization is influenced by factors such as the age of the mother, level of education of the women, occupational status of women and spouses, household economic status, place of delivery, birth order, awareness about obstetric related danger sign, and awareness about PNC services. However, factors influencing PNC services utilization vary from place to place [13, 14]. In sab Saharan Africa, various factors have been identified as significantly associated with postnatal care service use. Educational level of the mother, wealth quintile of the household, residence, gravidity, ANC visit, and place of delivery were identified as factors influencing postnatal care service utilization. But in sub-Saharan Africa, utilization of postnatal care service and associated factors are extremely variable and unsatisfying to design effective strategies [4, 12, 15].

In the previous studies, the logistic regression model was used, which cannot address a problem of non-independence [1, 3, 13, 16, 17]. But in this study, we used the multilevel model, which can handle both individual and community-level characteristics.

Moreover, there are many individual and community-level characteristics that are likely to influence postnatal care service utilization. Identifying, examining, and understanding these determinants is a first step in exploring strategies to reduce maternal mortality.

Therefore, a more advanced model, the multilevel logistic model was used to systematically identify and synthesize to quantify the pooled magnitude of postnatal care service utilization and determinants among reproductive-age women who gave live birth in sab Saharan Africa.

## Method

### Data source

The data used in this study were the most recent Demographic and Health Surveys (DHS) data compiled in the following sub-Saharan Africa countries (Angola, Benin, Burkina-Faso, Burundi, Cameroon, Chad, Comoros, Congo, Cote d’Ivoire, Democratic Republic of Congo, Ethiopia, Gabon, Gambia, Ghana, Guinea, Kenya, Lesotho, Liberia, Madagascar, Malawi, Mali, Mozambique, Namibia, Niger, Nigeria, Rwanda, Sao Tome & Principe, Senegal, Sierra Leone, Swaziland, Tanzania, Togo, Uganda, Zambia, Zimbabwe South Africa) from 2006 to 2018 (Table 1). These datasets were appended together to determine the pooled magnitude and determinants of postnatal care service utilization across the Sub-Saharan Africa countries. The DHS is a nationwide representative survey that collects data on basic health indicators like mortality, morbidity, family planning service utilization, fertility, maternal and child health. The data were derived from the measure DHS program. Each country’s survey consists of various datasets including men, women, children, birth, and household datasets, for this study IR file, was used.

**Table 1:** Pooled Demographic and Health Surveys (DHS) data from 36 sub-Saharan countries, 2006–2018

Country	DHS year	Sample size (286,255)
**Southern Region of Africa**		**9957**
Lesotho	2014	2575
Namibia	2013	3875
Swaziland	2006/07	514
South Africa	2016	3035
**Central Region of Africa**		**88,207**
Angola	2015/16	14,379
DR Congo	2013/14	18,827
Congo	2011/12	10,819
Cameroon	2011	15,426
Gabon	2012	8421
Sao Tome & Principe	2008/09	2615
Chad	2014/15	17,719
**Eastern Region of Arica**		**90,524**
Burundi	2010	8894
Ethiopia	2016	7590
Kenya	2014	6864
Comoros	2012	2064
Madagascar	2008/09	5395
Malawi	2015/16	13,469
Mozambique	2011	13,745
Rwanda	2014/15	2962
Tanzania	2015/16	7077
Uganda	2011	10,152
Zambia	2018	7324
Zimbabwe	2013/14	4983
**Western Region of Africa**		**97,567**
Burkina-Faso	2010	10,107
Benin	2017	9030
Cote d’Ivoire	2011	5223
Ghana	2014	4141
Gambia	2013	2060
Guinea	2018	5464
Liberia	2013	4769
Mali	2018	6604
Nigeria	2018	21,801
Niger	2012	8002
Sierra Leone	2010/11	8647
Senegal	2010/11	6864
Togo	2013/14	4850

The IR file contains all the collected data in the Woman’s Questionnaire for de facto women plus some variables from the Household Questionnaire. Up to 20 births in the birth history, and up to 6 children under age 5, for whom pregnancy and postnatal care, as well as immunization, health, and nutrition data, were collected, can be found as repeated variables in this file. This dataset use for most woman-level analysis including marriage and sexual activity, fertility, and fertility preferences, family planning, anthropometry and anemia in women, malaria prevention for women, HIV/AIDS, women’s empowerment, adult and maternal mortality, and domestic violence.

The DHS uses a two-stage stratified sampling technique to select the study participants. We pooled 36 DHS surveys done in the Sub-Saharan Africa countries and a weighted sample of 286,255 reproductive-age women who gave birth in the last 5 years preceding the survey was included in the study.

### Measurements of variables

#### Outcome variable

The outcome variable for this study was postnatal care services utilization. The variable is generated using WHO definitions of postnatal care services utilization which takes into account attendance of postnatal care checks by a health professional within 42 days of birth [1]. The outcome variable was binary and was coded as “1” if women got postnatal care service and”0″ otherwise.

#### Explanatory variables

Based on known facts and literature the independent variables: There are two types of variables considered for this study. The level one variable or individual-level variables and level two variables.

- **Level 1 or individual level** variable include maternal age, marital status, maternal education, occupational status, ANC visit, place of delivery, health care access, birth order, wealth index, and parity
- **Level 2 or community level variable** like country, residence, and Region (East, West, Central, and South)

### Data management and analysis

We pooled the data from the 36 sub-Saharan African countries together after extracting the variables based on literature. Before any statistical analysis, the data were weighted using sampling weight, primary sampling unit, and strata to restore the representativeness of the survey and take sampling design when calculating standard errors and reliable estimates. Cross tabulations and summary statistics were done using STATA version 14 software. The pooled prevalence of postnatal care service utilization with 95% Confidence Interval (CI) was reported for sub- Saharan Africa Countries from 2006 to 2018. Variables with *p*-value < 0.2 in the bi-variable analysis were considered in the multilevel logistic regression model. Adjusted Odds Ratios (AOR) with a 95% Confidence Interval (CI) and p-value ≤0.05 in the multilevel logistic model were used to declare significant factors associated with postnatal care utilization.

### Statistical modeling

For the determinants factors, the DHS data had a hierarchical structure, this violates the independence of observations and equal variance assumption of the traditional logistic regression model. Hence women are nested within a cluster and we expect that women within the same cluster may be more similar to each other than women in the rest of the country. This implies that there is a need to take into account the between cluster variability by using advanced models. Therefore, a multilevel logistic regression model (both fixed and random effect) was fitted. Since the outcome variable was binary, standard logistic regression and multilevel logistic regression models were fitted. In the multilevel logistic regression model, we ran four models to estimate both fixed effects of the individual and community-level factors and random intercept of between-cluster variation.

Empty model: the model analyzed without any factor variables, to test the random effect of between-cluster variability. Derived from the between-cluster and within-cluster variability, intra-class correlation coefficient (ICC) was estimated to determine if the data justified using a multilevel approach for analyses by depicting the magnitude of between-cluster variability.

Individual-level factors model: The second model examined effects of individual characteristics on postnatal care utilization. Besides, the ICC was estimated and observed if there was a decline in the between-cluster variability upon adding individual factors to the empty model.

Community-level factors model: This model contained only characteristics of clusters, not individuals. The unit of analysis for this model was the cluster.

Combined model: The important characteristics of individual women and clusters were concurrently fitted to one model to reveal their net fixed and random effects.

Model comparison and fitness were done based on the Intra-class Correlation Coefficient (ICC), Likelihood Ratio (LR) test, Median Odds Ratio (MOR), and deviance (−2LLR) values since the models were nested. The model with the lowest deviance was chosen.

Four models were fitted null model (models without the explanatory variables), the model I (models include community-level variables, model II (models include individual-level variable)), and Model III (models include both individual and community level variables) were fitted to select the best fit model for the data using LLR and Deviance. Model III which includes both individual and community level variable was selected because of its highest LLR and Smallest deviance (Table 3).

### Ethics consideration

Permission to get access to the data was obtained from the measure DHS program online request from http://www.dhsprogram.com.website and the data used were publicly available with no personal identifier.

## Result

In this study, 285,255 women who gave birth 5 years preceding the survey in 36 sub-Saharan Africa countries were included. Of these, the largest study participants 97,567(34.08%) were from Western Africa Region and the smallest study participants 9957(3.48%) were from Southern Regions of Africa. The majority of study participants 181,426(63.38%) were rural residents. The median age women included in his study was 28.8 (IQR = 7.2) years in which 117,219(40.95) of them under age category 25–34. Thirty-five percent of women and 36 % of men had no formal education. More than one-third of women 116,353(40.65) were under poor wealth status. Majority 255,498(89.26%) of women had antenatal care visits during their pregnancy. Seventy percent of women deliver their child at a health institution (Table 2).

**Table 2:** Distribution of postnatal service utilization in sub-Saharan Africa region

Variable	Postnatal care Utilization		Total (%)	X-square value	***p***-value
	Yes	No			
**Africa Region**					
Southern	6770	3187	9957(3.48)	98.41	< 0.001*
Central	57,053	31,153	88,207(30.81)		
Eastern	44,165	46,358	90,524(31.62)		
Western	48,641	48,924	97,567(34.08)		
**Residence**					
Rural	87,787	93,638	181,426(63.38)	104.44	< 0.001*
Urban	35,985	68,843	104,829(36.62)		
**Age group**					
15–24	56,853	39,426	96,279(33.63)	36.15	< 0.001*
25–34	58,506	58,713	117,219(40.95)		
35–46	41,271	31,485	72,756(25.42)		
**Maternal education**					
No education	46,935	56,073	103,008(35.98)	134.57	< 0.001*
Primary education	50,139	42,819	92,959(32.47)		
Secondary and above	59,555	30,731	90,287(31.54)		
**Husband education**					
No education	40,652	46,701	87,354(36.80)	30.12	< 0.001*
Primary education	32,590	32,569	65,160(27.45)		
Secondary and above	48,379	36,488	84,867(35.75)		
**Maternal Occupation**					
Had occupation	108,871	94,426	83,069(29.02)	196	< 0.001*
Had no occupation	47,871	35,198	203,185(70.98)		
**Wealth Index**					
Poor	55,828	60,524	116,353(40.65)	68.28	< 0.001*
Middle	30,789	25,907	56,696(19.18)		
Rich	70,012	43,192	113,205(39.55)		
**Media Exposed**					
Yes	115,797	74,269	190,246(66.47)	162.69	< 0.001*
No	40,647	55,341	95,968(33.53)		
**ANC visit**					
Yes	149,922	105,576	255,498(89.26)	87.35	< 0.001*
No	6690	24,042	30,732(10.74)		
**Place delivery**					
Home	28,337	53,439	81,777(29.17)	92.81	< 0.001*
Health Institution	122,415	76,179	198,594(70.83)		
**Wanted pregnancy**					
Yes	104,349	120,881	225,230(93.30)	4.19	0.041*
No	7455	8711	16,166(6.70)		
**Accessing health care**					
Big problem	91,608	81,345	172,954(60.70)	503.34	< 0.001*
Not big problem	63,776	48,015	111,791(39.26)		
**Birth Order**					
1	28,601	24,786	53,387(18.65)	28.11	< 0.001*
2–4	62,675	59,487	122,163(42.68)		
5+	65,354	45,350	110,704(38.67)		
**Birth weight**					
Low birth weight	18,532	24,517	43,050(17.83)	320.10	< 0.001*
Normal	93,265	105,090	198,355(82.17)		

*=indicates there is significant association between postnatal care and independent variable

### Pooled prevalence of postnatal care utilization

**Fig. 1:**
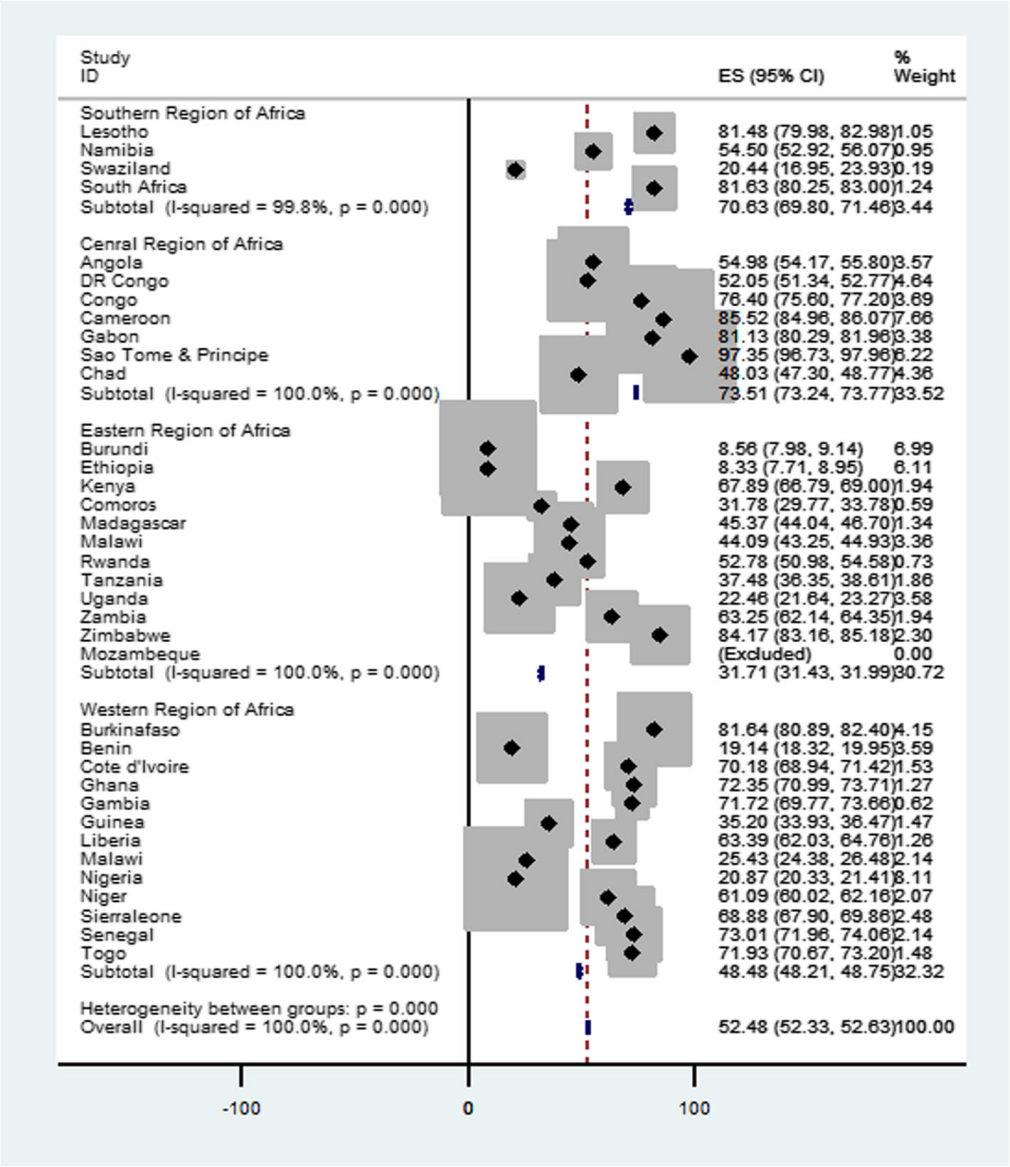
Forest plot of prevalence of postnatal care utlization in SSA countries

The pooled magnitude of postnatal care service utilization in sub-Saharan Africa countries was 52.48% [95% CI: 52.23, 52.63], with the highest postnatal care service utilization in central region of Africa (73.51%) and the lowest postnatal care service utilization in eastern regions of Africa (31.71%). The sub-group analysis result evidenced that in southern regions of Africa highest utilization of postnatal care service 81.48% were recorded in Lesotho and the smallest number of postnatal care service utilization 20.44% were recorded in Swaziland. In the central regions of Africa highest postnatal care utilization 85.52% were recorded in Cameroon and the lowest postnatal care service utilization 48.03% were from Chad. In Eastern regions of Africa highest postnatal care service utilization 84.17% were recorded in Zimbabwe and the lowest postnatal care service utilization 8.33% were from Ethiopia. In the western regions of Africa the highest postnatal care service utilization 81.64% were from Burkina Faso and the lowest utilization of postnatal care service utilization 19.14 were from Benin (Fig. 1).

### Determinants of postnatal care utilization

#### Random effect estimates

The model fitted for this study was multilevel multivariable logistic regression. There are two parts of estimates in this model. The random-effects estimates and fixed estimate. The random effect estimates were observed by fitting four models (Null model, Model I, Model II, Model III). The empty model shows that there was a significant variation in the likelihood of postnatal care utilization within sub-Saharan Africa Countries (ϭ^2^ = 0.31, *p* < 0.001). The ICC in the empty model implied that 8.66% of the total variation in postnatal care utilization was contributed to the difference between Countries. The cluster-level variance was expressed as ICC and MOR. Moreover, the MOR was 1.62 (95%CI:1.57,1.67) which implies that the odds of postnatal care service utilization was 1.62 times more likely when women go from low to high-risk countries. In model III (full model adjusted for individual and community level factors) cluster level variance (ϭ^2^ = 0.25, *p* < 0.001) remained significant but reduced. A total of 17.3% variability postnatal care utilization can be contributed to the country-level factors. The proportional change in variance (PCV) in this model was 17.30% which indicated 17.30% of the Country variance observed in the empty model was explained by both Country and individual level variable (Table 3).

**Table 3:** Multilevel multivariable logistic regression model result of postnatal care service utilization in sub-Saharan Africa from 2006 to 2018

Variable	Empty Model AOR(95%CI)	Community-level factors model AOR(95%CI)	Individual-level factors model AOR(95%CI)	Combined model AOR(95%CI)
**Africa Region**				
Southern		1		1
Central		0.78(0.74,0.81)		0.37(0.35,0.39)*
Eastern		0.51(0.49,0.54)		0.51(0.48,0.54)*
Western		0.49(0.47,0.52)		0.67(0.63,0.72)*
**Residence**				
Rural		1		1
Urban		1.72(1.69,1.75)		1.14(1.11,1.17)*
**Age group**				
15–24			1	1
25–34			1.10(1.07,1.13)	1.07(1.04,1.09)*
35–46			1.22(1.18,1.26)	1.15(1.12,1.19)*
**Maternal education**				
No education			1	1
Primary education			1.03(1.01,1.05)	1.15(1.12,1.18)*
Secondary and above			1.02(0.99,1.06)	1.11(1.07,1.14)*
**Husband education**				
No education			1	1
Primary education			0.89(0.87,0.91)	0.98(0.95,1.01)
Secondary and above			0.86(0.83,0.88)	0.97(0.95,1.02)
**Maternal Occupation**				
Had occupation			1	1
Had no occupation			1.03(1.01,1.06)	1.04(1.02,1.06)*
**Wealth Index**				
Poor			1	1
Middle			1.00(.97,1.02)	0.97(0.95,1.01)
Rich			0.98(0.96,1.01)	0.96(0.94,1.02)
**Media Exposed**				
No			1	1
Yes			1.85(1.82,1.89)	1.70(1.67,1.74)*
**ANC visit**				
No			1	1
Yes			3.03(2.95,3.13)	2.93(2.84,3.03)*
**Place delivery**				
Home			1	1
Health Institution			1.45(1.42,1.48)	1.45(1.42,1.48)*
**Wanted pregnancy**				
No			1	1
Yes			0.98(0.94,1.02)	1.00(0.96,1.04)
**Accessing health care**				
Big problem			1	1
Not big problem			1.05(1.03,1.07)	1.04(1.02,1.06)*
**Birth Order**				
1			1	1
2–4			0.93(0.90,0.96)	0.96(0.93,1.02)
5+			0.78(0.75,0.81)	0.97(0.95,1.03)
**Birth size**				
Low birth weight			1	1
Normal			1.08(1.05,1.11)	0.99(0.98,1.02)
**Random Effects results**				
Variance	0.31(0.27,0.35)	0.26(0.22,0.29)	0.24(0.21,0.27)	0.25(0.22,0.29)
ICC	8.66(7.76,9.66)	7.32(6.50,8.25)	6.83(6.03,7.73)	7.28(6.44,8.23)
PCV	1	16.67	22.75	17.30
MOR	1.70(1.60,1.75)	1.62(1.57,1.67)	1.59(1.54,1.64)	1.62(1.57,1.67)
**Model Comparison**				
LL	− 195,756	− 191,618	− 138,007	− 136,767
Deviance	391,512	383,236	2,760,014	273,534
AIC	391,517	383,248	276,052	273,581
BIC	391,538	383,311	276,247	273,817

*=significant at alpha 5%

#### The fixed effects analysis result

In the multilevel logistic regression model; Sub-Sahara Africa region, residence, age group, maternal education, maternal occupation, media exposure, ANC visit, Place of delivery, and accessing health care were statistically associated with postnatal care utilization in sub-Saharan Africa.

Women lining in central, eastern and, western Africa regions were decrease the likelihood of postnatal care service utilization by 63, 49 and 43% (AOR = 0.37, 95% CI: 0.35, 0.39) (AOR = 0.51, 95% CI: 0.48, 0.54) and (AOR = 0.67, 95% CI: 0.63, 0.72) as compared to women living in southern regions of Africa respectively. Women who reside in urban areas had 1.14(AOR = 1.14, 95% CI: 1.11, 1.17) times more likely to utilize postnatal care service than women living in rural areas. The odds of postnatal care service utilization among women of age group 25–34 and 35–49 were 1.07 and 1.15 time higher 1.07(AOR = 1.07, 95% CI: 1.04, 1.09) and 1.15 (AOR = 1.15, 95% CI: 1.12, 1.19) as compared to women age group 15–24 respectively. Women who had primary and secondary and above educational level had 1.15 (AOR = 1.15, 95% CI: 1.12, 1.18) and 1.11 (AOR = 1.11, 95% CI: 1.07, 1.11) times more likely to utilize postnatal care service than women who had no formal education. The odds of postnatal care service utilization among women who had occupations were increased by 4% as compared to women who had no occupation (AOR = 1.04, 95% CI: 1.02, 1.06). The odds of postnatal care service utilization was 1.70 (AOR = 1.70, 95% CI: 1.67, 1.74) times higher among women who exposed to media as compared to its counterpart. The odds of postnatal care service utilization among women who had antenatal care service visit during their pregnancy was 2.93 times higher as compared to women who had no ANC visit (AOR = 2.93, 95% CI: 2.84, 3.03). The odds of postnatal care service utilization among women who deliver their newborn from the health institution were 1.45 times higher as compared to women who deliver their baby at home (AOR = 1.45, 95% CI: 1.45, 1.48). The odds of postnatal care utilization were increased by 4% among women who reported accessing health care not a big problem as compared to its counterpart (AOR = 1.04, 95% CI: 1.02, 1.06) (Table 3).

## Discussion

This analysis was aimed at quantifying postnatal care service utilization and associated factors among sab Saharan countries women using the 2016 DHS data sets. Postnatal care has been approved to be an important maternal health service to keep and improve the health and survival of a mother and her newborn. Therefore, identifying the magnitude and factors of postnatal care may offer evidence for countries to reshape their policy directions.

This analysis discovered that 52.48% of women had utilized post-natal care service with a 95% confidence interval of 52.23 to 52.63%. This finding was lower than the 2013/14 DHS analysis report in Zambia (63%) [2]. (63%) (25). Though, this finding was higher than the 2016 DHS report 6.9% in Ethiopia [16], 2013 DHS 29% in Nigeria [18], 2015/2016 DHS 48.4% in Malawi [17], and a systematic review conducted in developing countries (36%) [12]. The possible reason for the observed difference might be occurred due to the existence of health system infrastructure and socio-cultural variations across countries. The possible reason for these differences might be due to several and complex factors, for instance, variations of policies against maternal health services, variability of quality of care and other factors like circumstances, value, understandings of postnatal care service [19].

Variables like residence, age group, maternal education, maternal occupation, media exposure, ANC visit, place of delivery, and accessing health care were statistically associated with postnatal care utilization in Sub-Saharan Africa.

Women who reside in an urban area had 1.14 times more likely to postnatal care utilization than women living in rural areas. This finding is supported by many other studies that showed a positive relationship between urban residency and postnatal care service utilization [2, 5, 9]. The discrepancy may be explained by physical proximity of health facilities, availability of better roads and transportation in urban than rural areas [20], and other possible causes of the discrepancy, maternal health services might be concentrated in urban areas than rural areas. Moreover, consciousness of maternal health services could be higher in urban areas than in rural areas.

The likelihood of postnatal care utilization among women of age group 25–34 and 35–49 were 1.07 and 1.15 time higher 1.07 and 1.15 as compared to women age group 15–24 respectively. These findings are supported a study conducted somewhere else [12, 17]. The possible explanation for this positive relationship might be because as women’s age increase the probability of health service experience on postnatal care will be better. Women who had primary and secondary and above educational levels had 1.15 and 1.11 times more likely to utilize postnatal care than women who had no formal education This finding agrees with other studies [5, 18, 21]. This can be due to as women become empowered, they could have information on advantages of postnatal care service utilization and they would be encouraged to have that service.

The odds of postnatal care utilization among women who had occupations were increased by 4% as compared to women who had no occupation This finding agrees with other reports elsewhere [17]. This can be explained as women have occupation the likely hood of being economic dependent decreases, as a result, they would have the chance to get the postnatal care service. The likelihood of postnatal care utilization was 1.70 times higher among women who exposed to media as compared to its counterpart. This finding is supported by other studies report somewhere else [4, 9]. This finding might be attributed to the fact that women who have access to media may tend to be aware of what complications they may confront when they fail to have postnatal visits. and use maternal health services to protect their health. The likelihood of postnatal care utilization among women who had antenatal care visit during their pregnancy was 2.93 times higher as compared to women who had no ANC visit. This finding is consistent with other findings reported somewhere else [6, 21]. This can be explained by the fact that during antenatal care service counseling, mothers could also be counseled on postnatal care services.

The odds of postnatal care utilization among women who deliver their newborn from the health institution were 1.45 times higher as compared to women who deliver their babies at home. This finding is in line with other reports elsewhere [2, 17, 22]. As mothers get deliver at health facilities, they are more likely to have counseling on postnatal care services and danger signs as well as exposed to health education. The odds of postnatal care utilization were increased by 4% among women who reported accessing health care not a big problem as compared to its counterpart This finding is consistent with another study in Nigeria [9]. This can be explained by the fact that women perceived that access to a health facility is not a big problem might be encouraged to have postnatal care services.

### Strength and limitation of the study

About the strengths, the dataset used in this study was obtained from nationally representative and the variables in the 36 Africa country DHS dataset were the same hence comparable across all countries. The study was population-based with a response rate of > 90%. The data were pooled together to create a large sample size of postnatal care service utilization reported within 5 years preceding each country survey which ranges from 2006 to 2018. It was able to identify the significant determinants of postnatal care service utilization across the 36 African Countries to inform policymakers and planners for their intervention to prioritize.

Regarding the limitations, the finding from this study may not establish a true causal relationship between the outcome variable and independent variables due to the cross-sectional nature of the study design. The data was collected based on self-report from mothers within 5 years preceding the survey and this could be a potential source of recall and misclassification bias.

## Conclusion

The coverage of postnatal care service utilization was low with high disparities among the region. Being a rural residence, young age group, low education level, had no occupation, not exposed to media, a big problem to access health care, not had ANC visit, and home delivery was associated with low postnatal care service utilization. This study evidenced that there is a wide gap in postnatal care utilization between SSA countries. Special attention is required to improve health accessibility, utilization, and quality of maternal health services to increase postnatal care service utilization in the region.

## Data Availability

Data is available online and you can access it from www.measuredhs.com

## Abbreviations

ANC: Antenatal Care
AOR: Adjusted Odds Ratio
CI: Confidence Interval
DHS: Demographic Health Survey
ICC: Intra-class Correlation Coefficient
LLR: Log-likelihood Ratio
LR: Likelihood Ratio
MOR: Median Odds Ratio
SSA: Sub-Saharan Africa
WHO: World Health Organization
